# Role of the CCAAT-Binding Protein NFY in SCA17 Pathogenesis

**DOI:** 10.1371/journal.pone.0035302

**Published:** 2012-04-17

**Authors:** Li-Ching Lee, Chiung-Mei Chen, Hao-Chun Wang, Hsiao-Han Hsieh, I-Sheng Chiu, Ming-Tsan Su, Hsiu-Mei Hsieh-Li, Chung-Hsin Wu, Guan-Chiun Lee, Guey-Jen Lee-Chen, Jung-Yaw Lin

**Affiliations:** 1 Department of Life Science, National Taiwan Normal University, Taipei, Taiwan; 2 Department of Neurology, Chang Gung Memorial Hospital, Chang-Gung University College of Medicine, Taipei, Taiwan; 3 Institute of Biochemistry and Molecular Biology, College of Medicine, National Taiwan University, Taipei, Taiwan; Emory University, United States of America

## Abstract

Spinocerebellar ataxia 17 (SCA17) is caused by expansion of the polyglutamine (polyQ) tract in human TATA-box binding protein (TBP) that is ubiquitously expressed in both central nervous system and peripheral tissues. The spectrum of SCA17 clinical presentation is broad. The precise pathogenic mechanism in SCA17 remains unclear. Previously proteomics study using a cellular model of SCA17 has revealed reduced expression of heat shock 70 kDa protein 5 (HSPA5) and heat shock 70 kDa protein 8 (HSPA8), suggesting that impaired protein folding may contribute to the cell dysfunction of SCA17 (Lee et al., 2009). In lymphoblastoid cells, HSPA5 and HSPA8 expression levels in cells with mutant TBP were also significantly lower than that of the control cells (Chen et al., 2010). As nuclear transcription factor Y (NFY) has been reported to regulate HSPA5 transcription, we focused on if NFY activity and HSPA5 expression in SCA17 cells are altered. Here, we show that TBP interacts with NFY subunit A (NFYA) in HEK-293 cells and NFYA incorporated into mutant TBP aggregates. In both HEK-293 and SH-SY5Y cells expressing TBP/Q_61∼79_, the level of soluble NFYA was significantly reduced. *In vitro* binding assay revealed that the interaction between TBP and NFYA is direct. HSPA5 luciferase reporter assay and endogenous HSPA5 expression analysis in NFYA cDNA and siRNA transfection cells further clarified the important role of NFYA in regulating HSPA5 transcription. In SCA17 cells, HSPA5 promoter activity was activated as a compensatory response before aggregate formation. NFYA dysfunction was indicated in SCA17 cells as HSPA5 promoter activity reduced along with TBP aggregate formation. Because essential roles of HSPA5 in protection from neuronal apoptosis have been shown in a mouse model, NFYA could be a target of mutant TBP in SCA17.

## Introduction

Spinocerebellar ataxia type 17 (SCA17) is an autosomal dominant ataxia caused by an expanded polyglutamine (polyQ) in a general transcription initiation factor, the TATA-box binding protein (TBP) [1], [2]. As a TATA-box recognition component, TBP and TAFs (for TBP-associated factors) form general transcription factor IID (TFIID) for RNA polymerase II to bind its promoter. In humans, the polyQ tract in TBP normally contains 25∼42 glutamine residues [3] and expanded alleles ranging from 43 to 66 glutamines have been shown to be associated with the disease [4], [5]. Expanded polyQ tracts enhanced the interaction of TBP with the general transcription factor IIB (TFIIB) [6]. In addition to progressive gait and limb ataxia, the broad phenotypic spectrum of this rare disorder includes seizure, cognitive dysfunctions, psychiatric symptoms, and pyramidal and extrapyramidal features such as spasticity, dystonia, chorea, and parkinsonism (review in [7]).

Protein misfolding and aggregation in the brain have been implicated as a common molecular pathogenesis of various neurodegenerative diseases. Our previously proteomics study using a cellular model of SCA17 has revealed reduced expression of heat shock 70 kDa protein 5 (HSPA5) [8], a major endoplasmic reticulum (ER) chaperone and master regulator of unfolded protein response (UPR) [9], suggesting that impaired protein folding in ER may contribute the cell dysfunction of SCA17. In lymphoblastoid cells, HSPA5 expression level in cells with mutant TBP was also significantly lower than that of the control cells [10]. Moreover, elimination of HSPA5 in Purkinje cells leads to accelerated cerebellar degeneration in a mouse model [11]. Thus investigating the regulation of HSPA5 expression in SCA17 cells may shed light on the pathogenesis of SCA17 and lead to development of therapeutics for the disease.

Nuclear transcription factor Y (NFY) is a unique evolutionarily conserved transcription factor that binds to CCAAT motifs in the promoter regions of a variety of genes. The trimeric NFY is formed by three subunits, NFYA, NFYB, and NFYC. The sequence specific interactions of the complex are made by the NFYA subunit, suggesting a role as the regulatory subunit [12], [13]. Recent studies focusing on NFY activity and heat shock 70 kDa protein 1A (HSPA1A) expression in the brain of a Huntington's disease (HD) mouse model have shown that mutant Huntingtin (Htt) aggregates sequester NFYA and NFYC leading to the reduction of HSPA1A gene expression, indicating NFY components as modulators of the HD pathological process [14]. Because NFY has been reported to regulate HSPA5 transcription [15], we investigated if NFY activity and HSPA5 expression are altered in SCA17 cells. We provided evidence to support the postulation that mutant TBP aggregates sequester NFYA to reduce functional NFY, leading to the reduction of HSPA5 gene expression. Considering the role of HSPA5 played in the SCA17 pathological process, therapeutic interventions for SCA17 may be developed through modulating NFYA expression.

## Results

### Incorporation of NFYA into mutant TBP aggregates in HEK-293 cells

**Figure 1:**
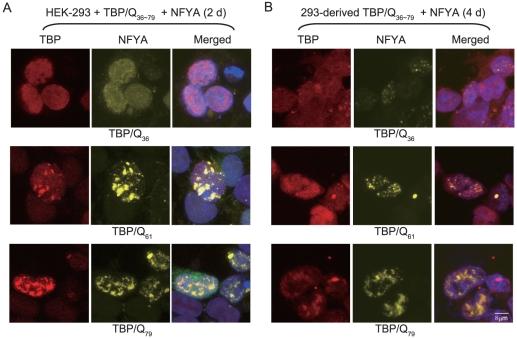
Localization of NFYA in TBP/Q_36∼79_ expressing 293 cells. (A) HEK-293 cells were transiently co-transfected with NFYA and TBP/Q_36∼79_. After 2 days, cells were fixed and stained with antibodies specific for TBP (red) and NFYA (yellow). (B) Isogenic 293 cells inducibly expressing TBP/Q_36∼79_ were transiently transfected with NFYA. After 4 days, cells were fixed and stained with antibodies specific for TBP (red) and NFYA (yellow). In both **a** and **b**, nuclei were counterstained with DAPI (blue). (The scale bar = 8 µm).

The trimeric NFY binds to CCAAT sequence to regulate gene transcription. In HD model mouse brain, mutant Htt reduces HSPA1A expression through sequestration of NFY components and reduction of NFY binding to HSPA1A promoter [14]. To determine whether NFYA, the regulatory subunit of NFY, incorporates into mutant TBP aggregates, HA-tagged TBP (normal TBP/Q_36_ and expanded TBP/Q_61_, TBP/Q_79_) and His-tagged NFYA constructs were transiently expressed in HEK-293 cells for 48 hr for immunocytochemical staining (TBP and NFYA) and fluorescence microscopy examination. As shown in Fig. 1A, while expressed TBP/Q_36_ was seen as diffuse nuclear staining, the expressed TBP/Q_61_ and TBP/Q_79_ protein formed aggregates and NFYA co-localized with TBP/Q_61_ and TBP/Q_79_ in nuclear inclusions. When 293-derived cells with inducible TBP/Q_36∼79_ expression for 96 hr and NFYA transfection were examined, positive nuclei with punctuate inclusions with NFYA co-localization were also visible in TBP/Q_61∼79_ cells (Fig. 1B).

### *In vivo* interaction between NFYA and TBP/Q_36∼79_

**Figure 2:**
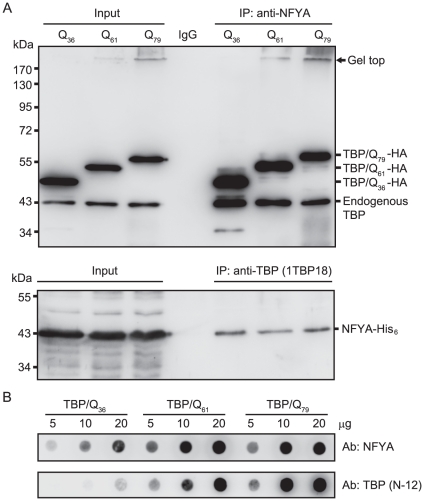
Interaction of NFYA and TBP/Q_36∼79_ *in vivo*. (A) HEK-293 cells were transiently co-transfected with NFYA and TBP/Q_36∼79_. After 48 h, cell lysates were prepared (Input, left panel) and immunoprecipitations (IP, right panel) were performed with anti-NFYA or anti-TBP (1TBP18) antibody. Rabbit or mouse IgG was used as a negative control for IP. Cell lysates and immunoprecipitates were analyzed with anti-TBP or anti-NFYA antibody. (B) After 48 h, insoluble pellets from NFYA and TBP/Q_36∼79_ co-transfected cells were lysed in SDS buffer and the indicated amounts of cell lysates (5∼20 µg) were trapped on an acetate membrane. The filter was probed with antibody against NFYA and TBP, respectively.

Next, a half-*in vivo* co-immunoprecipitation was performed to assess the intracellular association of NFYA with TBP. HEK-293 cells were co-transfected with HA-tagged TBP/Q_36∼79_ and His-tagged NFYA, and the expression of TBP and NFYA proteins were examined by Western blotting using anti-TBP and anti-NFYA antibodies. As shown in the left panel of Fig. 2A, the TBP antibody detected 47∼56 kDa HA-tagged TBP/Q_36∼79_ proteins in transfected cells, in addition to a small amount of endogenous 43 kDa TBP protein. While two variants of NF-YA which result from differential splicing was reported [16], the NFYA antibody detected a major 43 kDa His-tagged NFYA in transfected cells. The cell lysates were subjected to immunoprecipitation with anti-NFYA antibody and Western blotting with anti-TBP, or vice versa. As shown in the right panel of Fig. 2A, immunoblotting analysis showed that transfected HA-tagged TBP carrying 36∼79 glutamines were co-immunoprecipitated with NFYA, although endogenous TBP seems to be more capable of binding to NFYA. Similarly, transfected His-tagged NFYA was co-immunoprecipitated with TBP carrying 36∼79 glutamines. The presence of gel top bands in TBP/Q_61_ and TBP/Q_79_-expressing cells, but not in TBP/Q_36_-expressing cells, indicates the formation of SDS-insoluble aggregates by TBP/Q_61_ and TBP/Q_79_ and NFYA forms SDS-insoluble aggregates with mutant TBP. When the TBP/Q_36∼79_ and NFYA co-transfected samples were subjected to filter trap assay and stained with NFYA or TBP antibody, incorporation of NFYA into SDS-insoluble aggregates with TBP/Q_61_ and TBP/Q_79_ was significantly increased (Fig. 2B). The above data indicated that NFYA interacts with TBP carrying 36∼79 glutamines and NFYA is preferentially incorporated into SDS-insoluble aggregates with TBP/Q_61_ and TBP/Q_79_ in HEK-293 cells.

### *In vitro* interaction between NFYA and TBP/Q_36∼61_

Both glutathione S-transferase and thioredoxin soluble protein tags have been used in *E. coli* to increase recombinant protein expression level and/or solubility [17], [18]. Thus GST tag and Trx tag were fused to N-terminal TBP and NFYA respectively, for recombinant protein production in *E. coli*. The induced His-tagged GST-TBP/Q_36∼61_ (50∼56 kDa) and Trx-NFYA (60 kDa) fusion proteins were purified to apparent homogeneity from cell extracts and verified by Western blotting with anti-TBP and anti-NFYA antibodies (data not shown).

**Figure 3:**
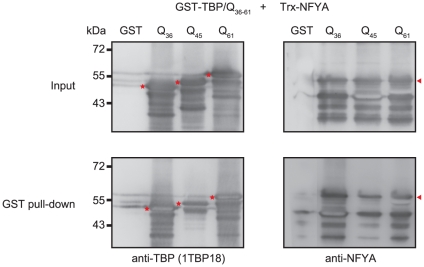
Interaction of NFYA and TBP/Q_36∼–61_ *in vitro*. His-tagged GST-TBP/Q_36∼61_ and Trx-NFYA fusion proteins were purified and incubated with glutathione agarose beads. Before (input) and after GST pull-down, each protein was detected by Western blot using anti-TBP and anti-NFYA antibodies. The positions of GST-TBP/Q_36∼61_ and Trx-NFYA were indicated by stars and an arrowhead, respectively.

To examine whether the *in vivo* interaction between TBP and NFYA is direct, we conducted a series of GST pull-down assay using the purified GST-His_6_-TBP/Q_36∼61_-His_6_ as bait protein and Trx-His_6_-NFYA-His_6_ as prey protein. This bacterial expression of both bait and prey proteins is commonly used to study protein-protein interaction [19], [20]. GST-His_6_-TBP/Q_36∼61_-His_6_ or GST-His_6_-His_6_ alone (as a negative control) was incubated with Trx-His_6_-NFYA-His_6_. Although the His tag allowed purification of the TBP and NFYA fusion protein to 90% homogeneity, undesirable proteolysis by a trace contaminant protease [21] generates various amount of cleaved products after GST pull-down. Apart from a light background band seen with GST alone, both wild type (36 glutamines) and expansion mutant (45 and 61 glutamines) TBP could pull down NFYA (Fig. 3). The data indicate that the recombinant Trx-His_6_-NFYA-His_6_ directly interacts with GST-His_6_-TBP/Q_36∼61_-His_6_.

### NFYA modulation of HSPA5 promoter activity

**Figure 4:**
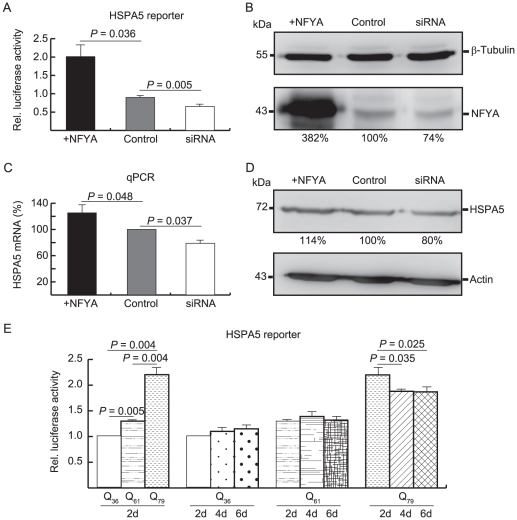
NFYA modulation of HSPA5 promoter activity. (A) HEK-293 cells were transiently transfected with HSPA5 reporter plasmid and luciferase activity measured two days post-transfection. Levels of luciferase activity in NFYA cDNA or siRNA co-transfected cells were expressed as folds of the activity of the HSPA5 reporter alone in HEK-293 cells (Control). (B) Cell lysates were prepared and analyzed with anti-NFYA or anti-β-tubulin antibody. (C) Real time PCR quantification of HSPA5 mRNA level relative to HPRT mRNA in HEK-293 cells transiently transfected with NFYA cDNA or siRNA for two days. Level of HSPA5 mRNA in vector-transfected cells were set as 100%. (D) Immunoblot analysis of HSPA5 protein level in HEK-293 cells transiently transfected with NFYA cDNA or siRNA for two days. Level of HSPA5 protein in vector-transfected cells were set as 100%. (E) 293-derived TBP/Q_36∼79_ cells were induced to express TBP for 2∼6 days and transiently express HSPA5 reporter plasmid for 2 days. Levels of luciferase activity were expressed as folds of the activity of the HSPA5 reporter in cells expressing TBP/Q_36_ for 2 days. All values are the mean ± SD of three independent experiments, each of which was performed in duplicate.

The proximal region of the human HSPA5 promoter contains three ER stress response elements consisting of CCAAT-(N)9-CCACG, in which CCAAT interacts with NFY [22]. Positive regulation of HSPA5 transcription by NFY has been shown in HeLa cells by Northern blot analysis [15]. HSPA5 promoter construct and NFYA cDNA were prepared and tested in HEK-293 cell transfection assay. As shown in Fig. 4A, luciferase level in cells co-transfected with NFYA cDNA and HSPA5 reporter plasmid was 198% (*P* = 0.036) of that in cells transfected with HSPA5 reporter plasmid alone. RNA interference assay was performed to examine the role of endogenous NFYA in regulation of HSPA5 gene transcription. Co-transfection of NFYA siRNA with HSPA5 reporter construct resulted in reduction in promoter activity (71%, *P* = 0.005). To ensure the over-expression and knock down of NFYA, immunoblot analysis was performed to assess the expression levels of NFYA. As shown in Fig. 4B, while a large amount (382%, *P* = 0.005) of NFYA was seen in cDNA-transfected cells, the amounts of NFYA were reduced (74%, *P* = 0.009) in siRNA-transfected cells.

To further evaluate if NFYA could modulate HSPA5 gene expression, real-time PCR and immunoblot analysis were performed to examine endogenous HSPA5 expression in cells transfected with NFYA cDNA or siRNA. As shown in Fig. 4C and 4D, 125% RNA (*P* = 0.048) and 114% protein (*P* = 0.033) were observed in cells transfected with NFYA cDNA and 79% RNA (*P* = 0.037) and 80% protein (*P* = 0.016) were seen in cells transfected with NFYA siRNA, when respectively compared with the control. Thus the results suggest that NFYA could modulate HSPA5 gene expression through the regulation of the promoter activity.

The incorporation of NFYA into mutant TBP aggregates in SCA17 cells raises the possibility that NFYA function was altered. To examine this, luciferase reporter assay to assess the HSPA5 expression was performed in 293-derived cells inducibly expressing normal TBP/Q_36_ or expanded TBP/Q_61_ and TBP/Q_79_. As shown in Fig. 4E, when luciferase activity for HSPA5 reporter was set to 100% in cells expressing TBP/Q_36_ for 2 days, luciferase activity driven by HSPA5 promoter in cells expressing TBP for 2∼6 days were 100∼115% for TBP/Q_36_ cells, 130∼139% for TBP/Q_61_ cells, and 187∼221% for TBP/Q_79_ cells. Compared to the cells expressing TBP for 2 days (TBP/Q_36_, 100%; TBP/Q_61_, 130%; TBP/Q_79_, 221%), while no significant change in HSPA5 promoter activity was seen in cells expressing TBP/Q_36_ (110∼115%, *P* = 0.098∼0.161) and TBP/Q_61_ (133∼139%, *P* = 0.243∼0.490) for 4 and 6 days, significant decrease of HSPA5 promoter activity was seen in cells expressing TBP/Q_79_ for 4 days (188%, *P* = 0.035) and 6 days (187%, *P* = 0.025). Together with the observed TBP aggregates with NFYA co-localization in 293-derived TBP/Q_79_ cells (Fig. 1B), the results suggest that the activation of HSPA5 transcription is altered in TBP/Q_79_ cells with TBP aggregates.

### NFYA overexpression to reduce TBP aggregation

**Figure 5:**
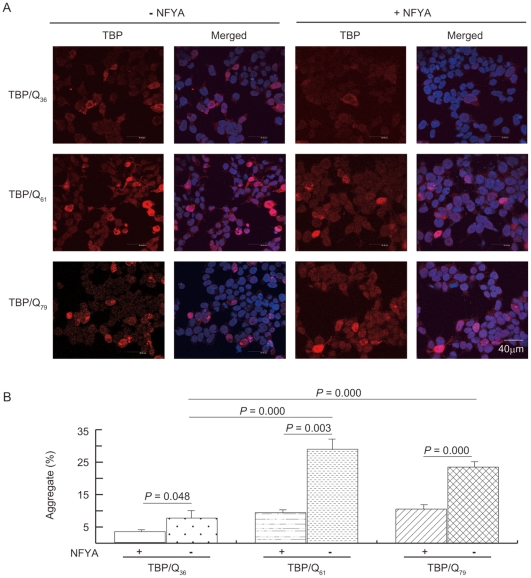
NFYA overexpression in SCA17 transient cell models. (A) HEK-293 cells were co-transfected with plasmids encoding TBP/Q_36_, TBP/Q_61_ or TBP/Q_79_, and plasmid with (+NFYA) or without (−NFYA) NFYA cDNA. After 2 days, cells were fixed and stained with TBP antibody (red). Nuclei were detected with DAPI (blue). (The scale bar = 40 µm) (B) The percentage of aggregate formation counted among five random fields.

To determine whether NFYA could suppress aggregation of mutant TBP, we transiently co-expressed NFYA with TBP/Q_36_, TBP/Q_61_ or TBP/Q_79_ in HEK-293 cells. Fig. 5A shows the images of fluorescence microscopy examination after immunostaining using TBP antibody (red). Without NFYA co-transfection, cells built visible aggregates were 7.8% for TBP/Q_36_ cells. Significantly increased visible aggregates were seen in TBP/Q_61_ cells (29.0%, *P* = 0.000) and TBP/Q_79_ cells (23.5%, *P* = 0.000). Co-transfection of NFYA effectively suppressed aggregate formation in TBP/Q_36_ (3.3% vs. 7.8%, *P* = 0.046), TBP/Q_61_ (9.4% vs. 29.0%, *P* = 0.003) as well as TBP/Q_79_ (10.5% vs. 23.5%, *P* = 0.000) cells (Fig. 5B).

### NFYA dysfunction in SCA17 cells

**Figure 6:**
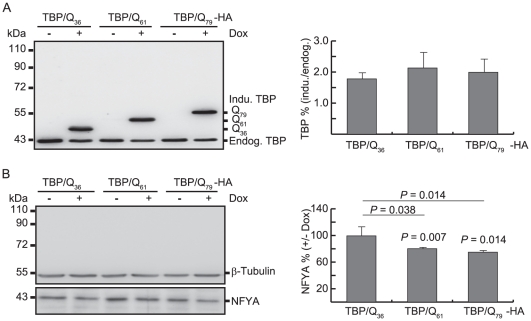
NFYA expression in TBP/Q_36∼79_ expressing 293 cells. (A) Western blot analysis of induced TBP protein level relative to endogenous TBP after 6 days induction (+Dox). (B) Western blot analysis of endogenous NFYA relative to endogenous β-tubulin after 6 days induction (+Dox).

To examine if expression of TBP/Q_61∼79_ suppressed the level of soluble NFYA protein, 293-derived cells with inducible TBP/Q_36∼79_ expression were examined. After six days induction, HA-tagged TBP-protein levels were first examined by Western blotting using TBP antibody. As shown in Fig. 6A, in addition to the 43 kDa endogenous TBP protein, the TBP antibody detected 47 kDa TBP/Q_36_-HA, 50 kDa TBP/Q_61_-HA and 53 kDa TBP/Q_79_-HA proteins in doxycycline induced TBP cells (178%∼214% of the endogenous TBP). In cells expressing TBP/Q_61∼79_-HA protein, a significant reduction of the endogenous soluble NFYA protein was observed compared to the non-induced TBP/Q_61∼79_ cells (80.1∼74.8% vs. 100%, *P* = 0.007∼0.014) or induced TBP/Q_36_ cells (80.1∼74.8% vs. 99.4%, *P* = 0.038∼0.014) (Fig. 6B).

**Figure 7:**
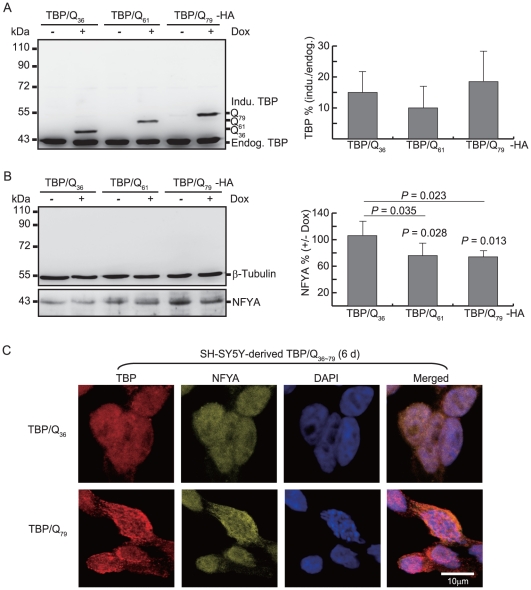
Inducible expression of TBP/Q_36∼79_ in SH-SY5Y cells. (A) Western blot analysis of induced TBP protein level relatively to endogenous TBP after 6 days induction (+Dox). (B) Western blot analysis of endogenous NFYA relatively to endogenous β-tubulin after 6 days induction (+Dox). (C) Isogenic SH-SY5Y cells were inducibly expressed TBP/Q_36∼79_ for 6 days. Cells were fixed and stained with antibodies specific for TBP (red) and NFYA (yellow), and nuclei were counterstained with DAPI (blue). (The scale bar = 10 µm).

The pcDNA5/FRT/TO-TBP constructs containing Q_36_, Q_61_ and Q_79_ were also used to generate isogenic SH-SY5Y TBP lines. After six days induction, TBP and NFYA levels and were examined by Western blotting using TBP and NFYA antibodies. Although levels of the induced TBP/Q_36∼79_-HA proteins were low (10.0%∼18.5%) compared to the endogenous TBP (Fig. 7A), in SH-SY5Y cells expressing TBP/Q_61∼79_-HA protein, a significant reduction of the endogenous soluble NFYA protein was observed compared to the non-induced TBP/Q_61∼79_ cells (76.2∼74.1% vs. 100%, *P* = 0.028∼0.013) or induced TBP/Q_36_ cells (76.2∼74.1% vs. 105.8%, *P* = 0.035∼0.023) (Fig. 7B). Positive nuclei with punctuate inclusions with NFYA co-localization were also detected in TBP/Q_61_ (data not shown) and TBP/Q_79_ cells (Fig. 7C).

## Discussion

Previous studies have suggested the involvement of transcriptional dysregulation, including increased Cre-dependent transcriptional activity, reduced TFIIB occupancy of the Hspb1 promoter, and reduced binding of TBP to DNA, in SCA17 pathogenesis [6], [23], [24]. In the present study, we identified NFY component A (NFYA) as a TBP aggregates-interacting protein in HEK-293 cells (Fig. 1 and Fig. 2). The reporter gene assay and examination of endogenous HSPA5 expression further indicated the positive regulation of NFYA on HSPA5 transcription (Fig. 4A∼4D). The result is consistent with the reported mutant TBP binding to NFY and inhibiting its association with HspA5 promoter to reduces HspA5 expression in SCA17 knock-in mice [25]. In addition, it has been shown that mutant Htt caused increased levels of reactive oxygen species (ROS) in neuronal and non-neuronal cells [26] and ER stress caused by ROS activated HSPA5 expression [27]. As TBP with a polyQ expansion significantly increased ROS generation and induced oxidative stress in 293 cells (data not shown), the marked increase of HSPA5 reporter activity in TBP/Q_79_ cells (221%, *P* = 0.004) (Fig. 4E) may be due to increased levels of ROS. Along with aggregate formation after 4∼6 days induction of TBP expression (Fig. 1B), HSPA5 transcription in TBP/Q_79_ cells was significantly decreased (Fig. 4E). These data suggest that the NFY complex loses its function due to sequestration of NFYA to mutant TBP aggregates, resulting in the reduction of HSPA5 gene transcription. The NFYA dysfunction was also supported by the reduced heat shock 70 kDa protein 8 (HSPA8, with two reverse CCAAT motifs) promoter activity along with TBP aggregate formation (data not shown). Indeed, previously reduced expression of HSPA8 has been observed in SCA17 cells [8], [10].

HSPA5 (also known as 78 kDa glucose-regulated heat shock protein GRP-78) is involved in the folding and assembly of proteins in the endoplasmic reticulum. Abnormal protein folding due to decreased rate of HSPA5 hydrolysis and disturbed SIL1-HSPA5 interaction is the cause of Marinesco-Sjogren syndrome that is characterized by ataxia, progressive myopathy and cataract [28]. In UPR pathway, NFY has been reported to control ER stress-inducible HSPA5 transcription to increase the protein-folding capacity of ER [15]. De-activation of HSPA5 transcription in TBP/Q_79_ cells by TBP aggregates may lead to impairment of protein folding, reduction of stress response, and induction of cell death. As HSPA5 overexpression leading to suppression of mutant TBP aggregation [8], reduced expression of HSPA5 by NFYA sequestration may further accelerate aggregation of mutant TBP. This would also induce incorporation of some other transcriptional factors into aggregates, which may result in altered expression of their target genes. These phenomena might be cooperating together to promote SCA17 progression.

In addition to de-activation of HSPA5 transcription, reduction of functional NFY may also lead to altered expressions of other NFY target genes. NFY interacts specifically with the CCAAT motif, one of the common promoter elements present in the proximal promoter of numerous mammalian genes transcribed by RNA polymerase II [12]. By cooperative interactions with other transcription factors that bind to a specific promoter, NFY regulates the transcription of various promoters [29], [30], [31], [32], [33], [34]. Thus expressions of NFY target genes other than HSPA5 might be also affected. In HD R6/2 mouse brain, reduced expression of HSP70 and 12 other genes containing more than two CCAAT sequences in their putative promoter regions might be caused by NFYA sequestration [14]. Further studies will be needed to understand the relationship between the NFY function and the other altered gene expressions in SCA17 models.

Unlike preferential interaction with aggregated form of mutant Htt in neuro2a cells [14], NFYA interacts with both soluble and SDS-insoluble TBP in HEK-293 cells (Fig. 2). Given the proximity of TATA-box and CCAAT-box, the two recognition-factors for these motifs are in close distance to each other. This may mimic direct interaction of TBP and NFYA and explain the advantage of endogenous TBP binding to NFYA (Fig. 2A). In HeLa cells, it has been shown that NFY controls ER stress-inducible transcription through recruitment of both ATF6(N) and TBP [15]. Using *in vitro* binding assay, we demonstrated that NFYA and TBP interact directly (Fig. 3). This NFYA-TBP interaction may facilitate the incorporation of NFYA into mutant TBP aggregates.

The transcription activation domain of NFYA is rich in glutamine and hydrophobic residues, and shows amino acid sequence similarity with the glutamine-rich activation domain of transcription factor Sp1 [12]. Since NFYA interacts physically with Sp1 *in-vitro* [29] and multiple NFY- and SP1-binding sites exist within HSPA5 proximal promoter region, SP1 may interact with NFY to regulate HSPA5 expression. In that context, SP1 could be another direct or indirect target of mutant TBP to induce the de-activation of HSPA5 promoter activity in SCA17 cells. Whether binding of SP1 to HSPA5 promoter DNA in SCA17 is reduced remains to be determined.

Although SH-SY5Y cells with full-length TBP can not be greatly induced, the SH-SY5Y cells with N-terminal TBP/Q_36∼79_ had been established with several fold transgene expression compared to endogenous TBP expression (data not shown). Like TBP, most polyQ disease proteins are widely expressed and some are critical for cellular function. However, the selective neurodegeneration is paradoxical. How the polyQ domain contributes to the normal function of proteins and the expanded polyQ induces selective neuropathology remain unclear. It is important that all the mechanisms proposed thus far provide explanations for the acceleration of neuronal dysfunction and/or cell death in specific neurons. We will continue our attempt to establish SCA17 neuron model for assessing the potential therapeutic targets underlying SCA17 pathogenesis.

In summary, we identified NFYA as a new TBP aggregate-binding protein and a probable modulator of the SCA17 pathological process. Further studies of the role of NFY in SCA17 pathology would reveal novel aspects of neuronal degeneration, leading to development of pharmacotherapeutics for the disease.

## Materials and Methods

### TBP and NFYA cDNA constructs

Polyadenylated RNA (200 ng) isolated from neuroblastoma SK-N-SH cells (ATCC No. HTB11) was reverse transcribed using the SuperScript™ III reverse transcriptase (Invitrogen). The TBP/Q_36_ cDNA in pGEM-T Easy vector (Promega) was constructed as described [8]. The TBP/Q_45_, TBP/Q_61_ and TBP/Q_79_ cDNAs were made by ligating *Fnu*4HI partially digested fragments and the repeat number was verified by DNA sequencing. The TBP containing 36, 61 and 79 glutamines were cloned into pEF-IRES/hrGFP vector as described [8]. The sense and antisense (His-tagged) primers used for nuclear transcription factor Y alpha variant 1 (NFYA, NM_002505) cDNA amplification were GGCTGGAGCCTCTGATTGGGTTTC and GTGGTGGTGGTGGTGGTGGGACACTCGGATGAT (His_6_ sequence underlined). The amplified 1.2 kb full-length cDNAs were cloned into pGEM-T Easy and sequenced. The cDNAs were then excised with *Eco*RI and subcloned into pcDNA3 (Invitrogen). The above TBP and NFYA constructs were used in transient expression study.

### Cell culture and transfection

Human embryonic kidney (HEK)-293 cells (ATCC No. CRL-1573) were cultivated in Dulbecco's modified Eagle's medium containing 10% fetal bovine serum in a 37°C humidified incubator with a 5% CO_2_ atmosphere. HEK-293-derived cells inducibly expressing TBP/Q_36∼79_ were constructed as described [8] and maintained in medium containing 5 µg/ml blasticidin and 100 µg/ml hygromycin. Doxycycline (10 µg/ml) was used to induce TBP expression. For transient overexpression, cells were plated into 6-well (6×10^5^/well) or 12-well (on coverslips, 2×10^5^/well) dishes, grown for 20 hr, and transfected using lipofectamine 2000 (Invitrogen) with TBP/Q_36∼79_ and/or NFYA cDNA plasmids (4 µg each/6-well or 2 µg each/12-well). The cells were grown for 48–96 hr for the following immunocytochemical staining (12-well dishes) and immunoprecipitation and dot-blot studies (6-well dishes).

### Immunocytochemical staining

Cells were washed with phosphate buffered saline (PBS) and fixed in 4% paraformaldehyde in PBS for 10 min, followed by 20 min incubation with 0.1% Triton X-100 in PBS to permeate cells, overnight incubation with 0.5% bovine serum albumin (BSA) in PBS to block non-specific binding. The primary antibodies TBP (N-12, Santa Cruz) and NFYA (H-209, Santa Cruz), diluted 1∶500 in 1% BSA in phosphate buffered saline (PBS), were used to stain cells at 4°C overnight. After washing, cells were incubated for 2 hr at room temperature in Cy5-conjugated secondary antibody (Zymed) diluted to 1∶500 in PBS containing 1% BSA, and washed with PBS. Nuclei were detected using 4′-6-diamidino-2-phenylindole (DAPI). The stained cells were examined for dual fluorescent imaging using a Leica TCS confocal laser scanning microscope.

### Immunoprecipitation and Western blotting

Total protein from TBP/Q_36∼79_ and NFYA co-transfected cells was prepared using buffer containing 50 mM Tris-HCl, 150 mM NaCl, 1 mM EDTA, 1 mM EGTA, 0.1% SDS and 0.5% sodium deoxycholate, 1% Triton X-100, protease inhibitor cocktail (Sigma). After sonication and centrifugation at 15000 rpm for 10 min, supernatants were incubated with anti-NFYA, anti-TBP (1TBP18, Abcam) or rabbit or mouse IgG (2 µg per 200 µg of total proteins in 200 µl reaction) for 60 min at 4°C, after which the antibody-protein complex were precipitated with Protein G Agarose (20 µl, Pierce), separated on 10% SDS-polyacrylamide gel electrophoresis (PAGE) and blotted onto nitrocellulose membranes by reverse electrophoresis. After blocking, the membrane was stained with antibody to TBP (N-12, 1∶3000 dilution) or NFYA (1∶500 dilution). The immune complexes were detected using horseradish peroxidase-conjugated goat anti-mouse or goat anti-rabbit (Jackson ImmunoResearch) IgG antibody (1∶10000 dilution) and chemiluminescent substrate (Millipore).

### Dot-blot filter retardation assay

Transfected cells were lysed on ice for 30 mins in buffer containing 50 mM Tris-HCl pH8.8, 100 mM NaCl, 5 mM MgCl_2_, 0.5% (w/v) NP40, 100 mM EDTA, and protease inhibitors cocktail. After centrifugation for 5 min at 14000 rpm, insoluble pellets were resuspended in buffer (20 mM Tris-HCl pH8.0, 15 mM MgCl_2_) containing DNase I (0.5 mg/ml). After 37°C incubation for 1 hr, the reaction was terminated by adjusting the mixture to 20 mM EDTA, 2% SDS and 50 mM DTT, followed by heating at 98°C for 5 min. Protein concentration was determined (Bio-Rad Protein Assay) using BSA as a standard. Extracted protein (5∼20 µg) were diluted into 2% SDS and filtered on a BRL dot-blot filtration unit through a cellulose acetate membrane (0.2 µm pore size, Schleicher and Schuell) pre-equilibrated with 2% SDS. Filters were washed twice in 0.1% SDS, blocked in TBS containing 3% nonfat dried milk and stained with the TBP (N-12) or NFYA antibody (1∶500 dilution). The immune complexes on the filter were detected as described above.

### Trx-tagged NFYA and GST-tagged TBP expression constructs

To generate Trx (thioredoxin)-tagged NFYA, the NFYA cDNA in pGEM-T Easy was excised with *Eco*RI and subcloned into *Eco*RI-digested pET-32b(+) (Novagen) for Trx-His_6_-NFYA-His_6_ expression in BL21(DE3)pLysS.

To generate GST (glutathione S-transferase)-tagged TBP, the N-terminal TBP (160 amino acids for TBP/Q_36_) was PCR amplified using the cloned TBP/Q_36∼61_ cDNA as a template and synthetic 5′ and 3′ primers GAACACCATGGATCAGAACAACAGCCTGCCAC and GGCTCGAGTGGCGTGGCAGGAGTGATGGGGGTC (*Nco*I and *Xho*I restriction sites underlined). After cloned into pGEM-T Easy vector and sequenced, the N-terminal TBP/Q_36∼61_ cDNAs were excised with *Nco*I and *Xho*I and subcloned into *Nco*I- and *Xho*I-digested pET-32b(+). In advance, the *Nde*I fragment containing Trx tag in pET-32b(+) was replaced with a *Nde*I fragment containing GST which was amplified by PCR using pGEX-5X-3 as a template and synthetic 5′ and 3′ primers GCATATGTCCCCTATACTAGGTTATTGG and GGCATATGACGACCTTCGATCAGAT (*Nde*I restriction site underlined).

### Protein purification and *in vitro* binding assay

Bacteria transformed with recombinant TBP/Q_36∼61_ or NFYA were grown in a liquid culture to an A_600_ of 0.6, and expression was induced with 0.1 mM isopropyl-β-D-thiogalactopyranoside (IPTG) for 2 hr at 37°C. Bacterial cells were then harvested and the His-tagged GST-TBP/Q_36∼61_ and Trx-NFYA were purified using His-Bind resins (Novagen) according to supplier's instructions.

In GST pull-down experiments, GST-tagged TBP/Q_36–61_ and Trx-tagged NFYA (10 µg each) were incubated with GST-Bind™ resin (50 µl, Novagen) in 600 µl of PBS containing 0.5% NP-40 (vol/vol) for 2 hr at 4°C. After centrifugation at 500× g for 5 min, pellets were washed twice with 1 ml of binding buffer. The proteins associated with the beads were separated on 10% SDS-PAGE and Western blotted with anti-TBP (1∶500 dilution) and anti-NFYA (1∶500 dilution) antibodies. The immune complexes were detected using horseradish peroxidase-conjugated goat anti-rabbit IgG antibody and chemiluminescent substrate as described.

### HSPA5 promoter construct and dual luciferase assay

The sense and antisense primers used for HSPA5 promoter (−608∼+14) amplification were ACGAACGGCCCTGAGACTCGCATA and ATCTGTCTGTGCTGTCTTGGCCGG. The amplified promoter fragment was cloned into pGEM-T Easy and sequenced. The cloned promoter fragments were inserted upstream of the firefly luciferase reporter gene in a dual luciferase reporter plasmid [35].

In experiments examining the role of NFYA in HSPA5 transcription, HEK-293 cells were plated into 6-well (6×10^5^/well) dishes, grown for 20 hr, and transfected with the HSPA5 dual luciferase reporter plasmid (4 µg). For cDNA co-transfection, equal amounts of HSPA5 reporter plasmid and NFYA cDNA plasmid (4 µg each) were employed. For siRNA co-transfection, 120 pmol of human NFYA siRNA (sc-29947, Santa Cruz) were used along with HSPA5 reporter plasmid (4 µg). The cells were grown for 48 h. Cell lysates were prepared and luciferase activity was measured with a luminometer using a dual luciferase assay system (Promega). The activity of each promoter was directly measured and expressed as the ratio of the firefly luciferase level to the *Renilla* luciferase level. Three independent transfection experiments were performed and difference in luciferase activity was tested using the two-tailed Student's *t*-test. To assess the expression and knock-down of NFYA, Western blotting using NFYA and β-tubulin (1∶5000 dilution, Sigma) antibodies was performed as described above.

In experiments examining the role of NFYA in SCA17 cells, 293-derived TBP/Q_36∼79_ cells were plated in 24-well dishes (5×10^4^/well). Next day, doxycycline was added for 2, 4 and 6 days to induce TBP expression. Two days before harvesting cells for luciferase assay, cells were transfected with HSPA5 reporter plasmid (1.5 µg).

### Real-time PCR and immunoblot analysis of HSPA5 expression

HEK-293 cells were plated into 6-well dishes and transfected with NFYA cDNA or siRNA as described. Forty-eight hours later, total RNA was extracted from cells using the Trizol (Invitrogen). The RNA was DNase treated, quantified, and reverse-transcribed to cDNA using the SuperScript™ III reverse transcriptase (Invitrogen). Using ABI StepOne™ Real-Time PCR System (Applied Biosystems), real-time quantitative PCR was performed on a cDNA amount equivalent to 12.5 ng total RNA with TaqMan fluorogenic probes Hs99999174_ml for HSPA5 and 4326321E for HPRT1 (endogenous control; Applied Biosystems). Fold change was calculated using the formula 2^ΔCt^, ΔC_T_ = C_T_ (HPRT1)−C_T_ (HSPA5), in which C_T_ indicates cycle threshold. Statistical analysis of differences between the groups was carried out using one-way analysis of variance (ANOVA).

For HSPA5 protein analysis, total protein was prepared 48 hours after NFYA cDNA or siRNA transfection. Protein was separated on 10% SDS-PAGE and blotted onto nitrocellulose membranes as described. After blocking, the membrane was stained with antibody to HSPA5 (1∶500 dilution, Santa Cruz) or actin (1∶5000 dilution, Millipore). The immune complexes were detected as described.

### SCA17 SH-SY5Y cell lines generation

The Flp-In™ T-REx™ System (Invitrogen) was used to generate stably induced SH-SY5Y cell lines exhibiting tetracycline-inducible expression of TBP/Q_36∼79_. Firstly SH-SY5Y-derived FIp-In host cells were generated from independent integration of plasmids pcDNA6/TR (a plasmid expressing the Tet repressor; selected with 5 µg/ml blasticidin) and pFRT/lacZeo (a plasmid containing the Flp Recombination Target (FRT) site; selected with 100 µg/ml Zocin) into the genome of SH-SY5Y cells (ATCC No. CRL-2266). Then the SH-SY5Y host cells were co-transfected with pOG44 plasmid (constitutively expressed the Flp recombinase) and pcDNA5/FRT/TO-TBP/Q_36∼79_ plasmid [8] according to the supplier's instructions. The repeats in these TBP cell lines were examined by PCR. These cell lines were grown in medium containing 5 µg/ml blasticidin and 100 µg/ml hygromycin. Doxycycline (dox, 5 µg/ml) was added to induce HA-tagged TBP expression for six days. The proteins were prepared for Western blotting using antibody to TBP (1TBP18, 1∶3000 dilution, Abcam), NFYA or β-tubulin as described.
